# Quality assessment of online patient information on upper gastrointestinal endoscopy using the modified Ensuring Quality Information for Patients tool

**DOI:** 10.1308/rcsann.2022.0078

**Published:** 2024-02-20

**Authors:** S Chien, GHL Miller, I Huang, DA Cunningham, D Carson, LS Gall, KS Khan

**Affiliations:** ^1^ NHS Greater Glasgow and Clyde, UK; ^2^ University of Glasgow, UK; ^3^ NHS Lanarkshire, UK

**Keywords:** Endoscopy, Oesophagogastroduodenoscopy, Upper gastrointestinal endoscopy, Modified Ensuring Quality Information for Patients tool, Patient information

## Abstract

**Introduction:**

Websites and online resources are increasingly becoming patients’ main source of healthcare information. It is paramount that high quality information is available online to enhance patient education and improve clinical outcomes. Upper gastrointestinal (UGI) endoscopy is the gold standard investigation for UGI symptoms and yet little is known regarding the quality of patient orientated websites. The aim of this study was to assess the quality of online patient information on UGI endoscopy using the modified Ensuring Quality Information for Patients (EQIP) tool.

**Methods:**

Ten search terms were employed to conduct a systematic review. for each term, the top 100 websites identified via a Google search were assessed using the modified EQIP tool. High scoring websites underwent further analysis. Websites intended for professional use by clinicians as well as those containing video or marketing content were excluded.

**Findings:**

A total of 378 websites were eligible for analysis. The median modified EQIP score for UGI endoscopy was 18/36 (interquartile range: 14–21). The median EQIP scores for the content, identification and structure domains were 8/18, 1/6 and 9/12 respectively. Higher modified EQIP scores were obtained for websites produced by government departments and National Health Service hospitals (*p*=0.007). Complication rates were documented in only a fifth (20.4%) of websites. High scoring websites were significantly more likely to provide balanced information on risks and benefits (94.6% vs 34.4%, *p*<0.001).

**Conclusions:**

There is an immediate need to improve the quality of online patient information regarding UGI endoscopy. The currently available resources provide minimal information on the risks associated with the procedure, potentially hindering patients’ ability to make informed healthcare decisions.

## Introduction

The general public’s access to health information has improved significantly over the past two decades owing to the evolution of the internet,^1^ with over 80% of internet users searching online for healthcare resources.^2^ As a result, there has been a dramatic increase in the number of websites providing healthcare information,^3^ allowing patients to access these resources before formally seeking professional advice.^4,5^ Although this may improve patient empowerment and involvement in healthcare decision making,^5^ such online resources can be unreliable, thereby adversely affecting patient health seeking behaviour.^6,7^ Additionally, inaccurate online information may contradict the evidence-based advice of healthcare professionals and could undermine the doctor–patient relationship, ultimately worsening patient outcomes.^8^ It is therefore crucial that the public can access high quality online health resources to improve patient education and expectations.

Given the impact that online resources may have on the informed consent and decision making process, it is important to ensure a reliable method of critical appraisal in order to analyse the quality of online patient information. The Ensuring Quality Information for Patients (EQIP) tool is a validated and reproducible means of evaluating written patient information,^9^ and it was recently expanded to include 36 items.^10^ This tool has previously been employed in the analysis of online patient information for a variety of medical conditions,^11–20^ highlighting its capabilities in providing a robust assessment of online patient resources.

Upper gastrointestinal (UGI) endoscopy is the gold standard test for investigation of UGI symptoms, allowing not only direct visualisation but also tissue biopsies and therapeutic intervention. Clinical demand for UGI endoscopy is continually increasing, with an estimated 3,000 UGI endoscopies performed per 250,000 of the population annually in the UK^21^ and over 7 million UGI endoscopies performed annually in the US.^22^

Obtaining informed consent is generally the responsibility of the endoscopist. However, patients are increasingly turning to the internet to access further information on UGI endoscopy in advance of their procedure. Consequently, high quality online patient resources are fundamental to improving patients’ understanding of UGI endoscopy, its indications, alternatives and associated complications, and yet evidence relating to the quality of online patient information for this procedure is sparse. The aim of this study was to assess the quality of online patient information on UGI endoscopy on top indexed websites using the modified EQIP tool.

## Methods

Data were gathered using the most popular online search engine, Google (www.google.com), forming a database for analysis. The terms “endoscopy”, “endoscopy procedure”, “oesophagogastroduodenoscopy (OGD)”, “gastroscopy”, “OGD procedure”, “upper GI endoscopy”, “gastrointestinal endoscopy”, “upper endoscopy”, “UGI endoscopy” and “gastroscopy procedure” were entered into Google. These terms were identified as commonly searched phrases using the AdWords™ Keyword Planner (https://ads.google.com/home/tools/keyword-planner/; Google, Mountain View, CA, US).

The first 100 websites for each search term were included in the analysis as most individuals limit searches to within the first 100 returned web pages.^18^ Duplicates were excluded from the database. All websites written in English and providing information intended for the general public on UGI endoscopy were considered eligible for analysis. Websites directed at both the adult and paediatric populations were included. Those intended for healthcare professionals were excluded as it was assumed that access to patients would be restricted and the use of medical jargon would impair patient understanding.^11^ Weblinks to video or marketing content and websites with information on colonoscopy only were also excluded. All data collection and analysis was undertaken over a four-week period between January and February 2021.

### Dataset creation

Each website was independently evaluated using the modified EQIP tool by four assessors (GM, IH, DC and DC), all of whom are fluent in English. Data entry was performed using Excel^®^ (Microsoft, Redmond, WA, US). The year and country of publication, and type of source (professional society, practitioner, patient group, academic centre, National Health Service [NHS] hospital, private hospital, government/health department, industry, news service, encyclopaedia) were also recorded. Repeat evaluation of each website was then carried out by a second assessor and any contradictory results were resolved by consensus.

### EQIP tool

All websites fulfilling the inclusion criteria underwent analysis using the modified EQIP tool.^12^ This resource utilises a 36-item checklist to evaluate the quality of patient information in three domains: content (items 1–18), identification (items 19–24) and structure (items 25–36). The content domain reviews whether sufficient medical information is incorporated in the resource. The identification domain looks at the degree to which the website displays production details. The structure domain analyses the readability and construction of the resource. In order to limit assessor subjectivity, all items were assessed by means of “yes/no” binary questions. The 75^th^ percentile was set as a cut-off point for the modified EQIP tool to discriminate high scoring websites from low scoring websites, as described in previous studies.^11–20^

### Additional items

Additional items outside the modified EQIP tool were included in the database to further appraise website quality. These included whether the website specifically discussed quantitative complication rates, risk of mortality, use of different sedation or anaesthetic techniques, emergency advice following the procedure and post-procedure recovery information for UGI endoscopy. Data collected were in the form of “yes/no” answers to minimise assessor subjectivity.

### Statistical analysis

The dataset comprised both continuous and categorical variables, reported as the median and interquartile range (IQR), and as numbers and percentages respectively. The Kruskal–Wallis test was employed to compare continuous variables, with Fisher’s exact test being utilised to compare proportions. All *p*-values were two-tailed. A *p*-value of ≤0.05 was deemed statistically significant.

## Results

**Figure 1:**
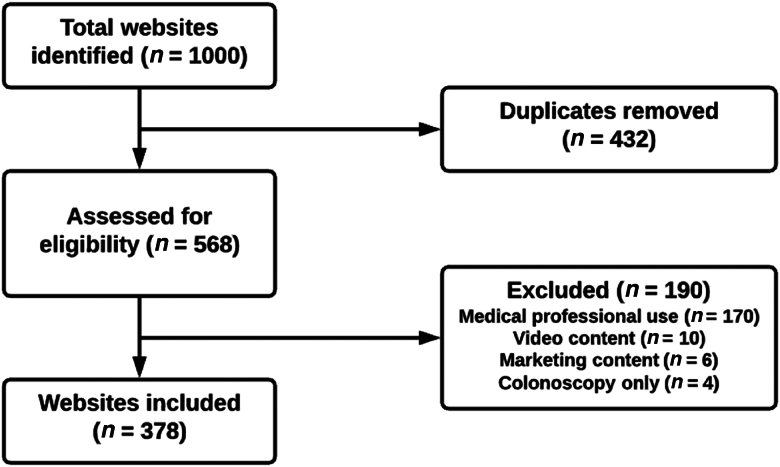
Workflow of the dataset creation

The initial dataset included 1,000 websites. Following removal of duplicate results that failed to meet the inclusion criteria, 378 websites remained eligible for analysis. The workflow for the creation of the dataset is shown in Figure 1. The initial website search was performed on a single date (25 January 2021) and evaluation of all websites was concluded within four weeks.

### Overall performance using the EQIP tool

**Figure 2:**
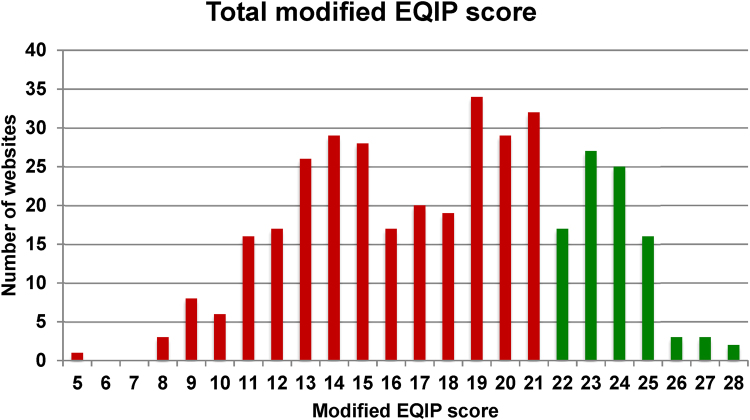
Distribution of modified Ensuring Quality Information for Patients (EQIP) scores for the included websites. The cut-off point for discriminating low scoring websites from high scoring websites was an EQIP score of 21 (75^th^ percentile).

The median modified EQIP score for all websites analysed was 18/36 (IQR: 14–21). The 75^th^ percentile cut-off was 21: all websites scoring ≥22/36 were deemed to be high scoring websites using the modified EQIP tool (*n*=93, 24.6%). The distribution of modified EQIP scores for all websites included in the analysis is shown in Figure 2.

**Figure 3:**
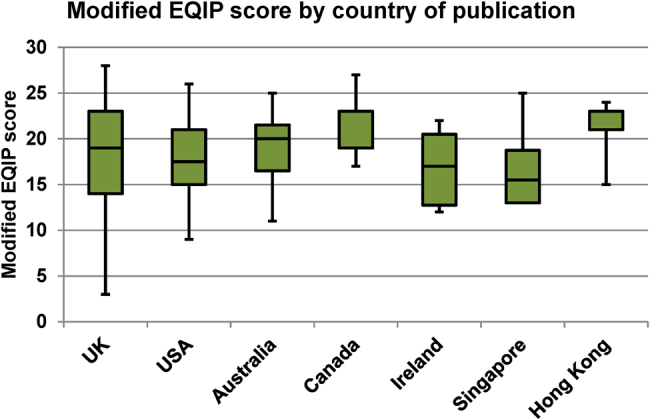
Median, interquartile range, minimum and maximum values for the modified Ensuring Quality Information for Patients (EQIP) score by country of publication with ≥5 websites (*p*=0.190)

The year of publication was available for 160 websites (42.3%), with 125 websites (33.1%) published within the past 5 years. Table 1 gives a breakdown of the country of origin and source of the websites. The returned websites originated from 13 countries. The UK produced the highest number of results (*n*=206), representing 54.5% of the total websites analysed. Fifty-eight of the UK websites were high scoring; this equated to 15.3% (58/378) of the websites overall and 62.4% (58/93) of the total high scoring websites. The modified EQIP score distribution by country of publication was scrutinised for all countries represented by ≥5 websites (Figure 3). There was no significant correlation between the modified EQIP scores and these countries (*p*=0.190).

**Table 1:** Number of websites included in the study grouped by country of origin and source of information (*n*=378)

	Number of websites
*Country*	
UK	206 (54.5%)
US	116 (30.7%)
Australia	19 (5.0%)
Canada	9 (2.4%)
Ireland	8 (2.1%)
Singapore	6 (1.6%)
Hong Kong	5 (1.3%)
India	3 (0.8%)
New Zealand	2 (0.5%)
Germany	1 (0.3%)
Greece	1 (0.3%)
Norway	1 (0.3%)
Poland	1 (0.3%)
*Source of website*	
Practitioner	123 (33.9%)
Hospital (National Health Service)	120 (31.7%)
Hospital (private)	47 (12.4%)
Government/health department	28 (7.4%)
Academic centre	21 (5.6%)
Patient group	16 (4.2%)
Professional society	12 (3.2%)
News service	9 (2.4%)
Encyclopaedia	2 (0.5%)

**Figure 4:**
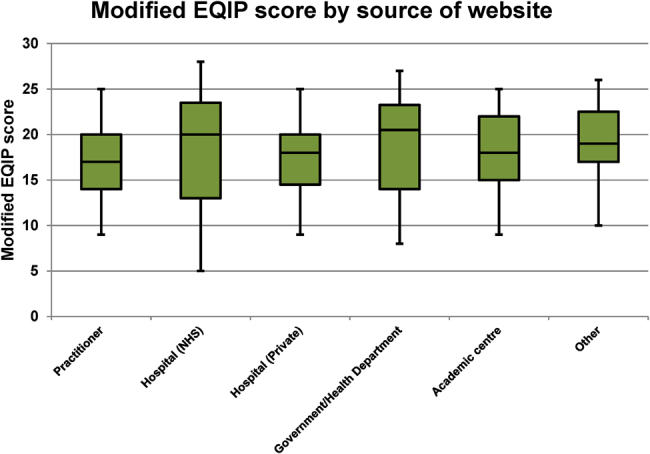
Median, interquartile range, minimum and maximum values for the modified Ensuring Quality Information for Patients (EQIP) score by source of website (*p*=0.007) NHS = National Health Service

Private practitioners were the most common source of website, accounting for 33.9% (123/378) of the database (Table 1). For further statistical analysis, the sources of the website were then categorised into “practitioner”, “hospital (NHS)”, “hospital (private)”, “government/health department”, “academic centre” and “other”. Figure 4 displays the distribution of modified EQIP scores by source of website. A statistically significant correlation was identified between website source and modified EQIP score (*p*=0.007). Government/health departments and NHS hospitals had the highest median modified EQIP scores (20.5/36 and 20/36 respectively). Among the total high scoring websites, 52.7% (49/93) originated from NHS hospitals.

### EQIP content domain

Table 2 summarises the results for the items comprising the content domain of the modified EQIP tool. The median modified EQIP score for content data was 8/18 (IQR: 5–10) (Table 3). No website achieved the maximum score available in this domain. High scoring websites were significantly more likely than low scoring websites to discuss the qualitative risks (97.8% vs 40.4%, *p*<0.001) and quantitative risks (55.9% vs 8.8%, *p*<0.001) of UGI endoscopy.

**Table 2:** Results for the content domain (items 1–18) of the modified Ensuring Quality Information for Patients tool

	Websites scoring points for domain items					
Content domain item	Overall (*n*=378)	High scoring (*n*=93)	Low scoring (*n*=285)	OR	95% CI	*p*-value
1. Initial definition of which subjects will be covered	69 (18.3%)	33 (35.5%)	36 (12.6%)	3.804	2.195, 6.594	**<0**.**001**
2. Coverage of the previously defined subjects	66 (17.5%)	33 (35.5%)	33 (11.6%)	4.200	2.402, 7.343	**<0**.**001**
3. Description of the medical problem/treatment/procedure	346 (91.5%)	93 (100%)	253 (88.8%)	–	–	**<0**.**001**
4. Definition of the purpose of the interventions	311 (82.3%)	91 (97.8%)	220 (77.2%)	13.443	3.223, 56.069	**<0**.**001**
5. Description of treatment alternatives (conservative management)	109 (28.8%)	63 (67.7%)	46 (16.1%)	10.911	6.376, 18.671	**<0**.**001**
6. Description of the sequence of the interventions and surgical procedure	303 (80.2%)	93 (100%)	210 (73.7%)	–	–	**<0**.**001**
7. Description of the qualitative benefits for the patient	290 (76.7%)	91 (97.8%)	199 (69.8%)	19.663	4.735, 81.651	**<0**.**001**
8. Description of the quantitative benefits for the patient	0 (0%)	0 (0%)	0 (0%)	–	–	1.000
9. Description of the qualitative risks and complications	206 (54.5%)	91 (97.8%)	115 (40.4%)	67.261	16.243, 278.516	**<0**.**001**
10. Description of the quantitative risks and complications	77 (20.4%)	52 (55.9%)	25 (8.8%)	13.190	7.388, 23.550	**<0**.**001**
11. Addressing quality of life issues	194 (51.3%)	79 (84.9%)	115 (40.4%)	8.342	4.507, 15.439	**<0**.**001**
12. Description of how complications are handled	112 (29.6%)	71 (76.3%)	41 (14.4%)	19.206	10.737, 34.355	**<0**.**001**
13. Description of the precautions that the patient may take	244 (64.6%)	92 (98.9%)	152 (53.3%)	80.500	11.067, 585.542	**<0**.**001**
14. Mention of alert signs that the patient may detect	132 (34.9%)	66 (71.0%)	66 (23.2%)	8.111	4.795, 13.719	**<0**.**001**
15. Addressing medical intervention costs and insurance issues	37 (9.8%)	5 (5.4%)	32 (11.2%)	0.449	0.170, 1.189	0.111
16. Specific contact details for hospital services	246 (65.1%)	64 (68.8%)	182 (63.9%)	1.249	0.757, 2.061	0.453
17. Specific details of other sources of reliable information/support	101 (26.7%)	36 (38.7%)	65 (22.8%)	2.138	1.296, 3.527	**0**.**004**
18. Coverage of all relevant issues for the topic (summary item for all content criteria)	28 (7.4%)	15 (16.1%)	13 (4.6%)	4.024	1.837, 8.814	**0**.**001**

CI = confidence interval; OR = odds ratio

### EQIP identification domain

The items for the identification domain of the modified EQIP tool are listed in Table 4. In the identification domain, the median modified EQIP score was 1/6 (IQR: 1–2) (Table 3). No website achieved the maximum score for this domain. High scoring websites were significantly better at providing the date of issue (89.2% vs 27.0%, *p*<0.001), the name of the website author (46.2% vs 11.2%, *p*<0.001) and a short bibliography (19.4% vs 4.2%, *p*<0.001).

**Table 3:** Analysis of modified Ensuring Quality Information for Patients (EQIP) scores obtained per domain

EQIP score	Total possible score	Median	Range	IQR
Content data	18	8	0–15	5–10
Identification data	6	1	0–5	1–2
Structure data	12	9	4–12	8–9
Total score	36	18	5–28	14–21

IQR = interquartile range

**Table 4:** Results for the identification domain (items 19–24) of the modified Ensuring Quality Information for Patients tool

	Websites scoring points for domain items					
Identification domain item	Overall (*n*=378)	High scoring (*n*=93)	Low scoring (*n*=285)	OR	95% CI	*p*-value
19. Date of issue or revision	160 (42.3%)	83 (89.2%)	77 (27.0%)	22.421	11.064, 45.433	**<0**.**001**
20. Logo of the issuing body	368 (97.4%)	92 (98.9%)	276 (96.8%)	3.000	0.375, 23.999	0.462
21. Names of the persons or entities that produced the document	75 (19.8%)	43 (46.2%)	32 (11.2%)	6.799	3.927, 11.773	**<0**.**001**
22. Names of the persons or entities that financed the document	1 (0.3%)	0 (0%)	1 (0.4%)	–	–	1.000
23. Short bibliography of the evidence-based data used in the document	30 (7.9%)	18 (19.4%)	12 (4.2%)	5.460	2.518, 11.838	**<0**.**001**
24. Statement about whether and how patients were involved/consulted in the document’s production	9 (2.4%)	4 (4.3%)	5 (1.8%)	2.517	0.662, 9.576	0.232

CI = confidence interval; OR = odds ratio

### EQIP structure domain

Table 5 shows the items for the structure domain of the modified EQIP tool. The median modified EQIP score for structure data was 9/12 (IQR: 8–9) (Table 3). Two websites (0.5%) achieved the maximum score (12/12) in this domain. Balanced information on the risks and benefits of UGI endoscopy was more readily available on high scoring websites (94.6% vs 34.4%, *p*<0.001).

**Table 5:** Results for the structure domain (items 25–36) of the modified Ensuring Quality Information for Patients tool

	Websites scoring points for domain items					
Structure domain item	Overall (*n*=378)	High scoring (*n*=93)	Low scoring (*n*=285)	OR	95% CI	*p*-value
25. Use of everyday language and explanation of complex words or jargon	364 (96.3%)	93 (100%)	271 (95.1%)	–	–	**0**.**026**
26. Use of generic names for all medications or products	359 (95.0%)	92 (98.9%)	267 (93.7%)	6.202	0.817, 47.110	0.053
27. Use of short sentences (<15 words on average)	366 (96.8%)	93 (100%)	273 (95.8%)	–	–	**0**.**044**
28. Personal address to the reader	317 (83.9%)	90 (96.8%)	227 (79.6%)	7.665	2.342, 25.093	**<0**.**001**
29. Respectful tone	377 (99.7%)	93 (100%)	284 (99.6%)	–	–	1.000
30. Clear information (no ambiguities or contradictions)	377 (99.7%)	93 (100%)	284 (99.6%)	–	–	1.000
31. Balanced information on risks and benefits	186 (49.2%)	88 (94.6%)	98 (34.4%)	33.584	13.202, 85.429	**<0**.**001**
32. Presentation of information in a logical order	373 (98.7%)	93 (100%)	280 (98.2%)	–	–	0.340
33. Satisfactory design and layout (excluding figures or graphs; see next item)	374 (98.9%)	92 (98.9%)	282 (98.9%)	0.979	0.101, 9.524	1.000
34. Clear and relevant figures or graphs	107 (28.3%)	50 (53.8%)	57 (20.0%)	4.651	2.820, 7.672	**<0**.**001**
35. Inclusion of a named space for the reader’s notes or questions	40 (10.6%)	15 (16.1%)	25 (8.8%)	2.000	1.005, 3.981	0.053
36. Inclusion of a printed consent form contrary to recommendations	12 (3.2%)	10 (10.8%)	2 (0.7%)	17.048	3.663, 79.346	**<0**.**001**

CI = confidence interval; OR = odds ratio

### Availability of additional information

Additional data were collected using the database to further investigate the reporting of quantitative complication rates, risk of mortality, use of different sedation or anaesthetic techniques, emergency advice following the procedure and post-procedure recovery information following UGI endoscopy. These data are summarised in Table 6.

**Table 6:** Websites reporting additional information

Additional information	Number of websites (*n*=378)
Sedation or anaesthetic information	297 (78.6%)
Post-procedure recovery information	221 (58.5%)
Emergency advice	132 (34.9%)
Quantitative complication rates	77 (20.4%)
Risk of mortality	22 (5.8%)

### Highest scoring websites according to the EQIP tool

The highest scoring websites (modified EQIP score ≥25/36) are shown in Table 7. Western Sussex Hospitals NHS Foundation Trust and Epsom and St Helier University Hospitals NHS Trust produced the joint top ranked resources, both scoring 28/36. These websites provide a comprehensive guide to UGI endoscopy, describing the procedure and its risks in detail, and providing date of production and contact details for hospital services. Interestingly, although these websites were top scoring, very few provided a formal bibliography and names of the website authors or addressed the cost of the procedure and insurance.

**Table 7:** Highest scoring websites (modified Ensuring Quality Information for Patients [EQIP] score ≥25/36)

Website author	Reference	Total EQIP score	Content data	Identification data	Structure data
Epsom and St Helier University Hospitals NHS Trust	23	28/36	14/18	3/6	10/12
Western Sussex Hospitals NHS Foundation Trust	24	28/36	13/18	3/6	12/12
Alberta Government	25	27/36	13/18	4/6	10/12
Hull University Teaching Hospitals NHS Trust	26	27/36	13/18	3/6	11/12
Royal Marsden NHS Foundation Trust	27	27/36	13/18	2/6	11/12
Alberta Government	28	26/36	12/18	4/6	10/12
MedicineNet	29	26/36	13/18	4/6	9/12
Northern Lincolnshire and Goole NHS Foundation Trust	30	26/36	13/18	3/6	10/12
Airedale NHS Foundation Trust	31	25/36	13/18	3/6	9/12
Bupa	32	25/36	12/18	3/6	10/12
Healthline	33	25/36	12/18	4/6	9/12
NHS Lothian	34	25/36	12/18	3/6	10/12
Oxford Radcliffe Hospitals NHS Trust	35	25/36	12/18	3/6	10/12
Queen Elizabeth Hospital King’s Lynn NHS Foundation Trust	36	25/36	13/18	2/6	10/12
Queensland Government	37	25/36	11/18	2/6	12/12
Royal Marsden NHS Foundation Trust	38	25/36	12/18	2/6	11/12
Royal United Hospitals Bath NHS Foundation Trust	39	25/36	13/18	2/6	10/12
Sandwell and West Birmingham NHS Trust	40	25/36	12/18	3/6	10/12
University of California San Francisco	41	25/36	13/18	3/6	9/12
Victorian Government	42	25/36	13/18	3/6	9/12
Gleneagles Hospital	43	25/36	12/18	3/6	10/12
Lahey Health	44	25/36	11/18	4/6	10/12
Verywell Health	45	25/36	12/18	3/6	10/12
West Suffolk NHS Foundation Trust	46	25/36	13/18	2/6	10/12

## Discussion

Given the increasing public access to the internet, the availability of comprehensive online patient information is crucial to the provision of patient centred care, with healthcare topics now contributing to the most frequently searched themes online.^47^ Although this improved accessibility of resources can be beneficial to patients, the lack of evidence-based peer review poses a threat to patients’ understanding and decision making related to their health conditions.^11^ As clinicians, it is vital to be able to direct patients towards online resources with sound reliability and credibility to supplement medical consultation.

The provision of high quality medical information to enhance patient understanding and education has been shown to improve clinical outcomes,^48^ and yet evidence focusing on the quality of websites related to UGI endoscopy is limited. A previous study analysed the quality and readability of just 45 websites providing information on UGI endoscopy using the Global Quality Score, Health On the Net certification, Flesch–Kincaid reading ease score and Flesch–Kincaid grade level.^49^ However, to our knowledge, our study is the first to examine online content intended for the general public on this procedure on a larger scale, having evaluated 378 patient information websites using the modified EQIP tool.

Various tools exist to gauge the quality of patient information, including the modified EQIP tool,^9,10^ the DISCERN tool^50,51^ and the *Journal of the American Medical Association* benchmark criteria.^52^ Although all three tools have been validated and are recognised internationally, the modified EQIP tool was chosen for this analysis as it provides an all-round assessment of health information, comprehensively appraising not only the content of information but also the format and readability of the websites.^9,10^ Furthermore, the modified EQIP tool has demonstrated high inter-rater reliability and reproducibility when used to evaluate information for a variety of other health conditions.^11–20^

Our analysis indicates that online patient information regarding UGI endoscopy is of limited quality, with a median EQIP score of 18/36 (50% of the maximum possible score). Additionally, the 75^th^ percentile cut-off score was low at 22/36 (61.1% of the maximum possible score). The lowest scoring domain was the reporting of identification data (median score: 1/6) while website structure scored highest (median score: 9/12). This suggests that although websites are largely coherent and user friendly, the reliability of the website content may be somewhat debatable given the lack of documented authorship and publication date on the majority of websites analysed. The production of websites to a higher visual standard is becoming easier as technology improves. This may present new challenges for those with limited clinical knowledge as websites with lower quality content may appear visually similar to resources with a high quality of information.

Although information was largely available on the procedure and purpose of UGI endoscopy, a concerning finding is the absence of balanced information on the risks and benefits in 50.8% of websites. Not only is this critical for the process of informed consent but it also enhances patient understanding of the rationale for the procedure, increasing patient cooperation and satisfaction.^53^

Quantitative complication rates and a description of how complications are handled were provided in only 20.4% and 29.6% of websites respectively. Despite being considered relatively safe, UGI endoscopic procedures are invasive and associated with a risk of iatrogenic perforation, which may ultimately require surgical intervention. It is evident that this unfavourable outcome may have a significant impact on the patient’s quality of life. Consequently, it is vital to provide information detailing the risks of the procedure in order to enable informed consent, as highlighted by previously published guidelines.^54^ The risk of mortality associated with UGI endoscopy is low; however, it is discouraging that this potential risk was only documented on 5.8% of websites. This should be considered standard practice when aiming to fully inform patients about the procedure.

Despite the fact that websites frequently underreported complication rates, it is worth noting that the majority of websites analysed were clearly intended to provide basic information prior to consultation with a clinician and it is therefore unrealistic to expect all websites to provide consent standard information. It is undoubtedly challenging to produce a website that covers all elements of UGI endoscopy comprehensively and we assume this may have contributed to the low median modified EQIP score overall.

High scoring websites generally demonstrated higher modified EQIP scores in the content domain. In particular, they were significantly more likely to provide patients with balanced information on the risks and benefits of the procedure (94.6% vs 34.4%, *p*<0.001), allowing patients to make better informed decisions. While high scoring websites scored significantly higher across 24 of the 36 EQIP items, a notable exception was the availability of medical insurance and intervention costs (5.4% vs 11.2%, *p*=0.111). This likely reflects the high proportion of websites from the UK, where medical treatment provided by the NHS is free.

In the identification domain, the date of publication and a bibliography were more readily available on high scoring websites, indicating that these websites utilise evidence-based approaches and are more likely to contain medically accurate information. The difference between high scoring and low scoring websites was less evident in the structure domain, suggesting that the majority of websites were readable and appropriate for distribution within the general population. Notably, 52.7% of the high scoring websites originated from NHS hospitals. This is useful for clinicians, who can be secure in the knowledge that they can direct patients towards these NHS websites as reliable online adjuncts to supplement clinical consultation.

The median modified EQIP score of 18/36 (IQR: 14–21) for UGI endoscopy is comparable with previous studies looking at various other medical conditions and procedures. These include gallstone disease (median EQIP score: 15, IQR: 13–18),^11^ liposuction (median EQIP score: 16, IQR: 14–18),^12^ bariatric surgery (median EQIP score: 17, IQR: 15–19),^14^ Dupuytren’s disease (median EQIP score: 16, IQR: 13–19),^15^ carpal tunnel syndrome (median EQIP score: 15, IQR: 13–17),^16^ breast augmentation (median EQIP score: 15, IQR: 12–20),^17^ living donor liver transplantation (median EQIP score: 16, IQR: 13–20),^18^ COVID-19 (median EQIP score: 18, IQR: 15–20)^19^ and acute appendicitis (median EQIP score: 20, IQR: 18–22).^20^

### Study limitations

This study had a number of limitations. Google was the only search engine employed as previous literature has shown that using multiple search engines merely generates duplicate results.^19^ Google search results are influenced by the geographical location of the requesting server as well as previous internet usage,^19^ suggesting that despite our comprehensive search strategy, the website results may not be wholly representative on an international level. This may also explain the high proportion of results from the UK in our analysis.

Additionally, our study included only English language websites. Consequently, our results may be of limited validity when applied to non-English language websites. Video content was also excluded as no tools are validated for the assessment of video-based information despite the fact that these can be valuable and easily understandable resources for patients with poor literacy. Finally, the internet is constantly expanding, with frequent addition of new websites and updates of existing pages. An evaluation of websites is therefore only representative of information available at a single timepoint.

## Conclusions

The internet is rapidly becoming patients’ principal source of information on healthcare topics and yet our study indicates that the quality of online patient information relating to UGI endoscopy is lacking, which could potentially have a significant impact on patients’ decision making. As clinicians, it is crucial for us to be able to confidently direct patients towards reliable online resources in order to improve patient education and clinical outcomes. For this reason, there is an immediate need for better quality online resources on UGI endoscopy.
